# Systemic administration of bone marrow‐derived cells leads to better uterine engraftment than use of uterine‐derived cells or local injection

**DOI:** 10.1111/jcmm.13294

**Published:** 2017-08-07

**Authors:** Ying Liu, Reshef Tal, Nicola Pluchino, Ramanaiah Mamillapalli, Hugh S. Taylor

**Affiliations:** ^1^ Department of Obstetrics, Gynecology & Reproductive Sciences Yale University School of Medicine New Haven CT USA; ^2^ Present address: Department of Reproductive Medicine Beijing Obstetrics and Gynecology Hospital Capital Medical University Beijing China

**Keywords:** stem cells, Asherman's syndrome, uterus, infertility, uterine stem cells

## Abstract

Stem cells are recruited to the uterus where they differentiate into endometrial cells and have been suggested as potential therapy for uterine injury such as Asherman's syndrome. However, it is unknown whether local intrauterine injection may result in better stem cell engraftment of the uterus compared with systemic administration, and whether uterine‐derived cells (UDCs) may confer an advantage over BM‐derived cells (BMDCs). Mice underwent local injury to a single uterine horn. Green fluorescent protein (GFP)‐expressing BMDCs, UDCs or saline (control) were injected either intravenously or locally (uterine lumen) into wild‐type recipients. Two or 3 weeks post‐transplant, uterine tissues were collected for fluorescence‐activated cell sorting (FACS) and immunohistochemistry/immunofluorescence studies. Mice injected intravenously with BMDCs or UDCs had increased GFP^+^ cells recruitment to the non‐injured or injured uterus compared to those injected locally. No significant differences were noted in GFP^+^ cell recruitment to the injured *versus* non‐injured horn. In addition, systemic injection of BMDCs led to greater recruitment of GFP^+^ cells at 2 weeks and 3 weeks compared with UDCs. Immunohistochemical staining demonstrated that GFP^+^ cells were found in stroma but not in epithelium or blood vessels. Immunofluorescence analysis revealed that GFP^+^ cells were mostly CD45‐negative, and negative for CD31 and cytokeratin, confirming their stromal identity. In conclusion, the systemic route of administration results in better recruitment of BMDCs or UDCs to the injured uterus than local injection. In addition, BMDCs recruitment to the uterus is greater than UDCs. These findings inform the development of stem cell‐based therapies targeting the uterus.

## Introduction

The uterine endometrium is a dynamic tissue containing glandular epithelium and stroma that undergoes regeneration in each reproductive cycle. The human endometrium undergoes more than 400 cycles of regeneration, differentiation and shedding during a woman's reproductive years 1, 2. It is comprised of two zones including the basalis and the functionalis layers. The functionalis layer undergoes destruction and regeneration with each menstrual cycle, which is necessary for human reproductive function. Disorders of endometrium have been implicated in abnormal uterine bleeding, endometriosis, endometrial cancer, infertility, miscarriage and pregnancy complications.

Endometrial stem cells are thought to reside in the basalis layer and serve as a source of progenitors that differentiate to form the endometrium. These endogenous progenitor stem cells facilitate the rapid replacement of the endometrial functionalis layer with each menstrual cycle. Endometrial stem cells have been differentiated into endometrial glandular epithelial, stromal and endothelial cells *in vitro* 3, 4, and in immunodeficient mouse models 4, 5, 6. In addition to these progenitor cells, there is a population of multipotent stem cells that reside in the uterus. Moreover, endometrial stem cells have been shown to have considerable plasticity, being able to differentiate into adipose, cartilage, muscle, cardiomyocytes and neurons 7, 8, 9, 10, 11, 12.

Adult bone marrow is a reservoir of stem and progenitor cells. Bone marrow‐derived cells (BMDCs) have the ability to transdifferentiate into multiple nonhematopoietic cell lineages including skin, muscle cells, neurons, hepatocytes, cardiomyocytes and gastrointestinal epithelium 13, 14, 15. BMDCs play a role in the reconstitution of the human endometrium. We and others have previously shown that BMDCs engraft the endometrium in rodents and humans, where they become epithelial, stromal as well as endothelial cells 16, 17, 18, 19.

Clinically, in infertile women, therapeutic uterine injury has been increasingly utilized in an attempt to improve endometrial receptivity and implantation in patients with recurrent implantation failure and/or thin endometrium 20, 21. We have previously shown that ischaemic/reperfusion injury provides a strong stimulus for homing and engraftment of BMDCs into the uterus 18, and it has been suggested that one of the mechanisms by which uterine injury may improve endometrial receptivity is *via* increasing recruitment of BMDCs to the endometrium.

Bone marrow‐derived cells have been shown to undergo recruitment into the uterus where they can differentiate into endometrial cells. Most animal models examining this phenomenon utilized bone marrow transplantation *via* systemic administration. We have shown that systemic administration of BMDCs can improve uterine scar healing and fertility in Asherman's syndrome mouse model 22. Recently, small clinical trials assessed the potential therapeutic effect of BMDCs in Asherman's syndrome in women following either systemic or intrauterine administration 23, 24. However, it is not known whether local intrauterine injection may result in better stem cell recruitment to the uterus compared with systemic administration. In addition, it is unknown whether UDCs may confer an advantage over BMDCs. This study was aimed at investigating and comparing the recruitment of BMDCs and UDCs into the endometrium following intra‐uterine injection or systemic administration after local injury.

## Materials and methods

### Animals and experimental groups

Transgenic C57BL/6J mice expressing enhanced GFP (UBC‐GFP) were obtained from Jackson Laboratory (Bar Harbor, ME, USA) Jand used as bone marrow or uterine cell donors. Wild‐type C57BL/6J female mice were obtained from Charles River Laboratories (Wilmington, MA, USA) and used as recipients of bone marrow or uterine cells injection. All animals were maintained in the Animal Facility of Yale University School of Medicine. Mice were housed 4–5 per cage in an animal room exposed to a 12‐hrs light/dark cycle (7:00 a.m.–7:00 p.m.) with food and water provided *ad libitum*. All animals were treated under an approved Yale University institutional animal care and use committee protocol.

### Uterine injury model

Prior to BMDCs/UDCs injection, 8‐week‐old C57BL/6J female mice (*N* = 84) were subjected to mild uterine injury according to our prior protocol 22 with minor modification. Briefly, after administration of isoflurane (Isothesia; Henry Schein, Columbus, OH, USA), a vertical incision was made in the abdominal wall and the uterus was exposed under sterile conditions. A 27 Gauge needle was inserted into the left horn lumen at the utero‐tubal junction, rotated and withdrawn four times gently.

**Figure 1:**
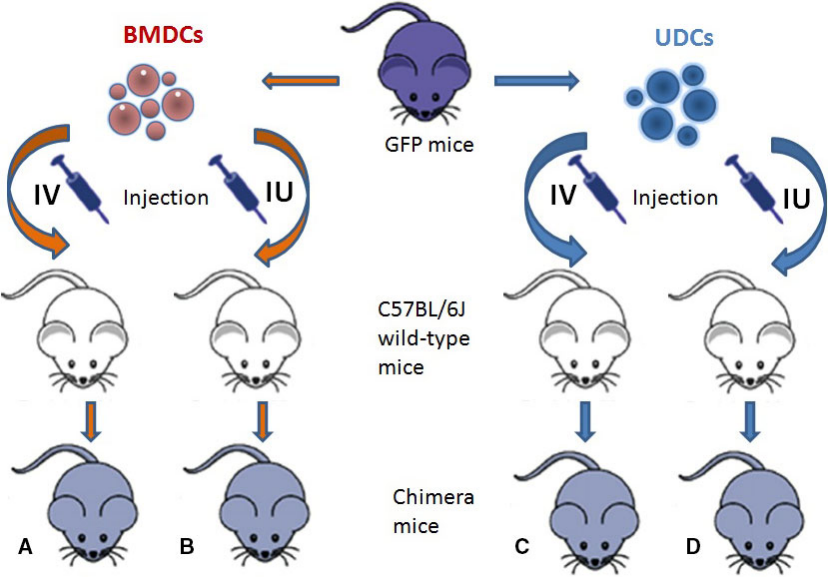
A schematic depicting the different regimens of systemic administration or local injection of BMDCs/UDCs in a mouse endometrial injury model. After induction of uterine injury of the left horn, mice received different treatment regimens of BMDCs or UDCs either intravenously (IV) or intrauterine into the lumen (IU).

Eight‐week‐old C57BL/6J wild‐type female mice received four different treatment regimens of BMDCs or UDCs as shown in Figure 1. Following induction of experimental uterine injury in the left horn, mice were divided into four treatment groups (*n* = 14 in each group). Group A (BMDCs‐iv) received 1 × 10^7^ BMDCs in 100 μl saline by tail vein injection; the local administration group B (BMDCs‐iu) was injected with 1 × 10^7^ BMDCs in 20 μl saline into the uterine lumen. Group C (UDCs‐iv) received 5 × 10^5^ UDCs in 100 μl saline by tail vein injection while group D (UDCs‐iu) was injected with 5 × 10^5^ uterine cells in 20 μl saline into the uterine lumen. Additionally, groups PBS‐iv and PBS‐iu received 100 μl saline by tail vein injection or 20 μl saline by uterine lumen injection, serving as controls.

### Bone marrow cells isolation and transplantation

Donor bone marrow cells were flushed from the femurs and tibias of 6‐ to 8‐week‐old C57BL/6‐Tg UBC‐GFP with cold sterile DMEM/F12 (Life Technologies, Carlsbad, CA, USA). The bone marrow cell suspension was filtered through sterile 70 μM Nitex mesh strainer, washed and resuspened with phosphate‐buffered saline. A total of 1 × 10^7^ unfractionated BM cells were injected either intravenously or into the uterine lumen of 8‐ to 10‐week‐old female Bl/6 recipients as described above.

### Endometrial derived cells isolation and transplantation

Donor uterine cells were obtained from the uteri of 6‐ to 8‐week‐old C57BL/6‐Tg UBC‐GFP female mice. Uterine horns were minced followed by enzymatic digestion with in Hanks' balanced salt solution (Life Technologies, Carlsbad, CA, USA) containing HEPES (25 mM), collagenase B (1 mg/ml; Roche Diagnostics, Indianapolis, IN, USA) and deoxyribonuclease I (0.1 mg/ml; Sigma‐Aldrich, St. Louis, MO, USA) for 60 min. at 37°C to yield single cell suspension. Cells were subsequently filtered through 40 μm mesh and centrifuged at 720g, 4°C for 5 min., followed by washing twice with cold sterile PBS and then re‐suspended in sterile PBS for injection. A total of 5 × 10^5^ total uterine cells were injected either intravenously or into the uterine lumen of 8‐ to 10‐week‐old female Bl/6 recipients as described above. In preliminary experiments conducted to determine the number of UDCs cells for injection, mice receiving 1 × 10^6^ uterine cells in 100 μl saline by tail vein injection showed some instances of mortality. Therefore, we chose a dose of 5 × 10^5^ cells for UDCs injections.

### Tissue harvesting

Recipient mice were euthanized at either 2 or 3 weeks post‐transplant. Mice were sacrificed by CO_2_ inhalation followed by cervical dislocation. Animals were perfused with saline to remove circulatory blood prior to extraction of the uterus. For each mouse, a part of each uterine horn was subjected to flow cytometry analysis, while the other part of the horn was fixed in 4% paraformaldehyde for further immunohistochemistry and immunofluorescence studies.

### Flow cytometry analysis of uterus

For flow cytometry analysis of GFP‐positive cells, uterine horn tissues were minced followed by enzymatic digestion with 1 mg/ml of collagenase B (Roche Diagnostics) and 0.1 mg/ml deoxyriboneclease (Sigma‐Aldrich) in Hanks' balanced salt solution (Life Technologies) in Hanks' balanced salt solution (Life Technologies) for 60 min. at 37°C. Cells were collected by filter through 40 μm mesh followed by centrifugation (720g at 4°C for 5 min.), washed twice with cold PBS and then re‐suspended in PBS. Flow cytometry was performed on a FACS Beckman Coulter MoFlo machine (Beckman Coulter, San Jose, CA, USA) using the corresponding excitation wavelength for GFP. Gates were applied to forward‐scatter/side‐scatter dot plots to exclude nonviable cells and cell debris. The percentage of positive cells was calculated against the background set on a fluorescence‐minus‐one negative control. Data were analysed using the software FlowJo V10 (Tree Star, Ashland, OR, USA).

### Immunohistochemistry and immunofluorescence

Uterine tissues for histological analysis were fixed in 4% paraformaldehyde for 16–24 hrs (overnight) at room temperature, dehydrated and then embedded in paraffin. Five micrometer sections were cut and mounted on slides, deparaffinized in xylene, rehydrated in graded ethanol washes and then stained with H&E for assessment of endometrial histology. For immunohistochemistry, antigen retrieval was performed with 0.01 M sodium citrate (pH: 6.0), endogenous peroxidase was blocked by adding 0.3% hydrogen peroxide (H_2_O_2_) (Sigma Chemical Co.) and incubating in a water bath at 37°C for 30 min. Samples were blocked with 10% goat serum albumin for 30 min. Sections were then incubated with rabbit anti‐GFP antibody (Abcam, Cambridge, MA, USA) (1:1000 dilution) overnight at 4°C. Incubation without primary antibody served as negative control. Samples were then incubated for 1 hr with biotinylated goat anti‐rabbit Ig antibody (Vector Biolabs, Burlingame, CA, USA) secondary antibody. Slides were treated with 3,3′ diaminobenzidine tetrahydrochloride (Sigma Chemical Co.) with TBS and 0.3% (H_2_O_2_) and counter‐stained with haematoxylin. Photomicrographs of the sections were taken using an Olympus BX41 microscope.

Immunofluorescence was used to perform colocalization studies of GFP^+^ cells in the uterus. Blocking was applied with 10% donkey serum (Sigma‐Aldrich) in PBS for 60 min. at room temperature. After blocking, sections were incubated overnight at 4°C with either of the following primary antibodies: polyclonal goat anti‐GFP (1:1000), rat anti‐CD45 (1:300), rabbit anti‐CD31 (1:200), rabbit anti‐vimentin (1:300) and rabbit anti‐pancytokeratin (1:200) (all from Abcam). The following secondary antibodies were used: AlexaFluor 564‐conjugated donkey anti‐goat and AlexaFluor 488‐conjugated donkey anti‐rabbit or AlexaFluor 488‐conjugated donkey anti‐rat (all diluted in 1:200) (Life Technologies) for 1 hr at room temperature. Nuclear counterstaining was performed by incubating slides with 4,6‐diamidino‐2‐phenylindole (DAPI) (Vector Laboratories, Burlingame, CA, USA). Negative controls excluding primary antibody were included in every staining. All the visualizations of the slides were performed with a laser scanning confocal microscope (LSM 710; Zeiss, New York, NY, USA) and the ZEN software (Carl Zeiss).

### Image capture and cell counting

For quantitative analysis of GFP^+^/CD45^+^ cells in the uterus, three random sections of slides from each mouse were captured at 400× magnification. Regions of endometrium were randomly chosen and full‐thickness was counted. Images were obtained by adequate excitation and emission filter sets: DAPI for nuclei, fluorescein isothiocyanate for GFP, rhodamine for CD45, CD31, cytokeratin and vimentin. They were subsequently analysed using Image J software (version 1.32j) (National institutes of Health, Bethesda, MA, USA). The total number of DAPI^+^ cell nuclei was counted, and the number of GFP^+^/CD45^+^ cells was then counted and expressed as a percentage of the total cell nuclei counted for per section. At least 1000 cells were counted per each animal.

### Statistics

Data were analysed using GraphPad Prism 6.0 software (GraphPad Software, Inc., La Jolla, CA, USA). Results are reported as median (range) or mean ± S.E.M. for each group. As the data were normally distributed (Kolgomorov–Smirnov normality test), one‐way anova and Holm–Sidak *post hoc* test for pairwise comparisons were undertaken for assessment of differences between groups. *P*‐values <0.05 were considered to be statistically significant.

## Results

### Histological evidence of no‐fibrosis in mouse uterine injury model

**Figure 2:**
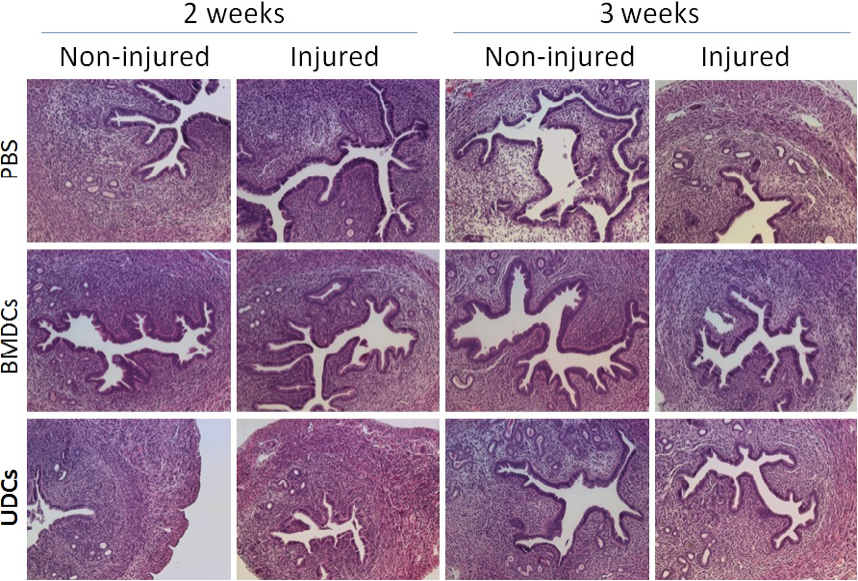
H&E histological sections of uteri at 2 or 3 weeks after uterine injury and BMDCs/UDCs injection showing normal endometrial histology without fibrosis. (Original magnification, ×100).

To confirm that our uterine injury model consisted of only mild endometrial injury and was not associated with scarring or fibrosis, the uterine horns were collected for histological assessment at 2 or 3 weeks following uterine injury and BMDC or UDC injection. Histological analysis showed normal endometrium without evidence of fibrosis in all mice groups (Fig. 2). In addition, no significant difference in histology of the uterus at 2 and 3 weeks after uterine injury was observed between BMDCs transplantation, UDCs transplantation and the control group based on H&E staining. Uterine injury was previously reported to stimulate BMDCs recruitment to the uterus 18. As the endometrial injury employed herein was mild, we wished to investigate whether it still results in increased recruitment of BMDCs to the uterus. A five fold increase in BMDCs recruitment to the uterus at 2 weeks post‐transplant was noted in the mice that underwent uterine injury to one horn as compared to sham control (*P* < 0.0001) (data not shown).

### Localized uterine injury recruits BMDCs / UDCs throughout the endometrium

To investigate the effects of single horn endometrial injury on engraftment of BMDCs or UDCs to uterus, both the injured and the non‐injured horns were collected at 2 and 3 weeks after BMDCs or UDCs transplantation and analysed for GFP^+^ cells by flow cytometry. For BMDCs‐iu injection, the GFP^+^ cell percentage in the non‐injured horn and injured horn was 0.042% *versus* 0.045% (*P* = 0.824) at 2 weeks, 0.03% *versus* 0.058% (*P* = 0.261) at 3 weeks; for BMDCs‐iv injection, the GFP^+^ cell percentage in the non‐injured horn and injured horn was 0.264% *versus* 0.261% (*P* = 0.970) at 2 weeks, 0.217% *versus* 0.22% (*P* = 0.969) at 3 weeks. For UDCs‐iu injection, the GFP^+^ cell percentage in the non‐injured horn and injured horn was 0.044% *versus* 0.0425% (*P* = 0.939) at 2 weeks, 0.02% *versus* 0.022% (*P* = 0.846) at 3 weeks; for UDCs‐iv injection, the GFP^+^ cell percentage in the non‐injured horn and injured horn was 0.022% *versus* 0.044% (*P* = 0.079) at 2 weeks, 0.0225% *versus* 0.048% (*P* = 0.051) at 3 weeks. FACS analysis showed no significant differences in GFP^+^ cell recruitment to the injured and non‐injured horn for both cell types at either time point, suggesting that the injury to one horn recruits BMDCs/UDCs to the uterus globally and not just the injured side**.**

### BMDCs engraft the uterus better than UDCs

**Figure 3:**
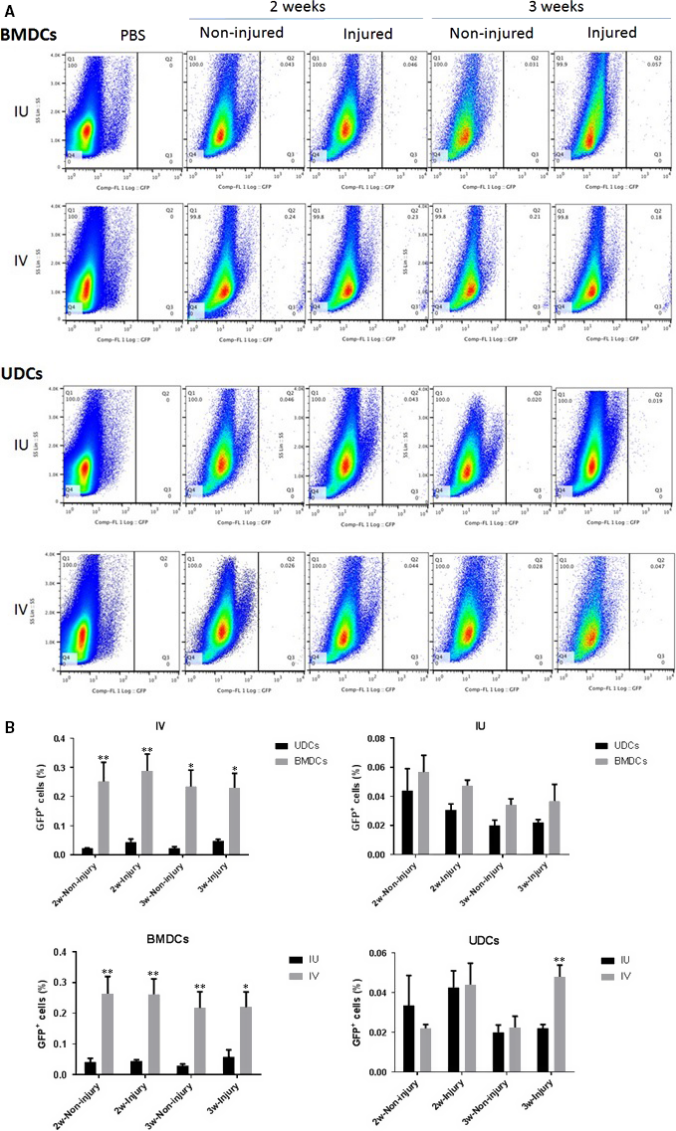
BMDCs engraft the uterus in greater numbers than UDCs and systemic administration results in better recruitment than local injection. Flow cytometry analysis of donor BMDCs or UDCs in the uterus. (**A**) Representative images of flow cytometry analysis of GFP‐positive cells after systemic administration or local injection of BMDCs or UDCs demonstrating the percentage of GFP‐positive cells in the uterus in the various regimens at 2 and 3 weeks post‐injection. (**B**) Mean %GFP‐positive cells at 2 and 3 weeks after systemic administration or local injection of BMDCs or UDCs. Data in graphs are presented as mean ± S.E.M.; *P < 0.05 *versus* other group; **P < 0.005 *versus* other group.

To compare the recruitment of BMDCs and UDCs to the uterus following injury, BMDCs and UDCs were injected to the recipient mice after the uterine injury, and uterine horns were analysed 2 or 3 weeks later. Flow cytometry analysis showed that GFP^+^ cell recruitment to the uterus following systemic injection was significantly greater with BMDCs as compared to UDCs injection (Fig. 3). At 2 weeks, the GFP^+^ cell percentage in the BMDCs‐iv group and UDCs‐iv group was 0.252% *versus* 0.022% (*P* = 0.004) in the non‐injured horn, and 0.288% *versus* 0.044% (*P* = 0.005) in the injured horn, respectively. At 3 weeks, the GFP^+^ cell percentage in the BMDCs‐iv group and UDCs‐iv group was 0.217% *versus* 0.0225% (*P* = 0.024) in the non‐injured horn, 0.22% *versus* 0.048% (*P* = 0.016) in the injured horn, respectively. In addition, following local injection, there were no significant differences between UDCs and BMDCs at 2 or 3 weeks post‐injection. No GFP signal was detected in control mice.

### Systemic administration of BMDCs / UDCs results in better uterine recruitment than local injection

Systemic administration of BMDCs resulted in increased recruitment of GFP^+^ cells to the non‐injured horn at 2 and 3 weeks compared to local injection (0.264% *versus* 0.042%, *P* = 0.008, 2 weeks) (0.217% *versus* 0.03%, *P* = 0.004, 3 weeks) (Fig. 3). Similarly, BMDCs injected systemically had increased recruitment of GFP^+^ cells to the injured horn at 2 and 3 weeks compared to those injected locally (0.261% *versus* 0.045%, *P* = 0.002, 2 weeks) (0.22% *versus* 0.058%, *P* = 0.029, 3 weeks).

Moreover, mice injected intravenously with UDCs demonstrated greater recruitment of GFP^+^ cells to the injured uterus at 3 weeks compared with those injected locally (0.048% *versus* 0.022%) (*P* = 0.003), although no significant differences were observed at 2 weeks. However, mice injected intrauterine with UDCs showed decline of GFP^+^ cells in the injured horn at 3 weeks compared with those at 2 weeks (0.022% *versus* 0.044%, *P* = 0.048).

### Localization of GFP^+^ cells in the uterus

**Figure 4:**
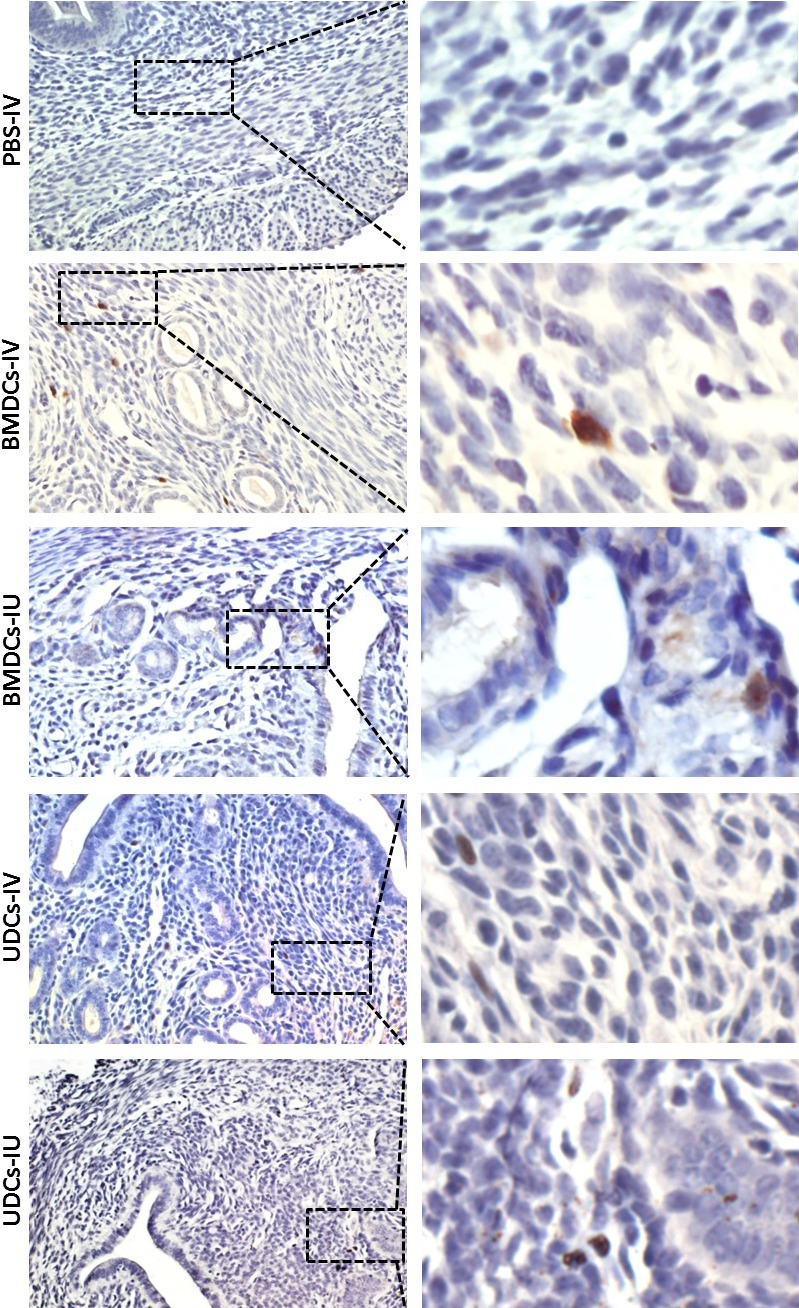
BMDCs or UDCs recruitment to the uterus after IV or IU injection. Immunohistochemistry staining of uterine sections using anti‐GFP antibody (brown) showing GFP‐positive cells in uterus in the various BMDCs or UDCs injection regimens. GFP‐positive cells give rise to stromal cells but not endothelial or epithelial cells in the uterus and tended to appear in perivascular location (left, original magnification x 400; right, higher magnification of the dashed rectangles).

To investigate the localization of the GFP^+^ cells which engrafted to the uterus of recipient mice, uterine sections were analysed by immunohistochemistry with GFP antibody. Immunohistochemical staining of uterine tissues demonstrated that GFP^+^ cells were found in myometrium and endometrial stroma but not in epithelium (luminal or glandular) or blood vessels (Fig. 4). Moreover, the stromal GFP^+^ cells tended to appear in perivascular location around both large and small vessels. There was no difference in terms of localization between BMDCs injection and UDCs injection groups (Fig. 4).

### BMDCs / UDCs engraft the uterus in the injury mouse model

**Figure 5:**
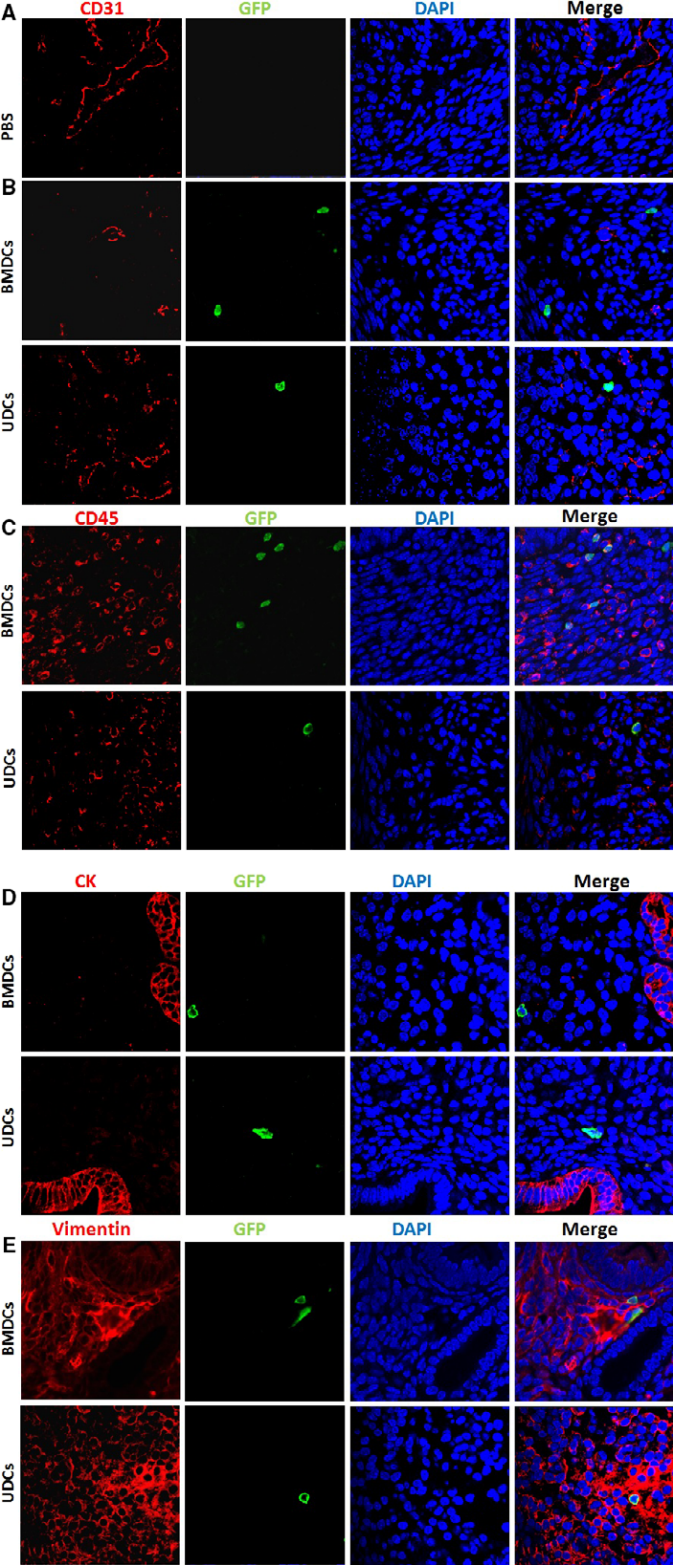
Engraftment of the endometrium with BMDCs or UDCs following systemic or local injection. Fluorescence confocal microscopy analysis of uterine tissue sections (**A‐E**). Uterine tissues of PBS control (**A**) or treated mice (**B‐E**) were stained with anti‐GFP antibody (green) and costained with either CD31 (endothelial marker) antibody (red) (**A** and **B**), anti‐CD45 (pan‐leucocyte marker) antibody (red) (**C**), cytokeratin (epithelial marker) antibody (red) (**D**) or vimentin (stromal marker) antibody (red) (**E**). Nuclei were stained by DAPI and are shown in blue. GFP‐positive cells did not colocalize with CD31 (**B**) or cytokeratin (**D**) markers, but colocalized with CD45 (**C**) and vimentin (**E**) markers. (magnification ×630).

In order to characterize the GFP^+^ cells engrafted to the uterus, we performed immunofluorescence colocalization analysis. We used CD45 as a pan‐leucocyte cell marker to distinguish non‐hematopoietic cells from leucocytes in the endometrium. In uteri of mice injected with BMDCs, 47.4% of endometrial stromal GFP^+^ cells were CD45 negative. In uteri of mice injected with UDCs, 64.3% of endometrial stromal GFP^+^ cells were CD45 negative. None of the control mice injected with PBS showed GFP‐positive staining. In addition, uterine sections were assessed for colocalization of GFP with CD31 (endothelial marker), vimentin (stromal marker) or cytokeratin (epithelial marker) as shown in Fig.5. Rare donor‐derived GFP positive cells were found to be vimentin positive, but cytokeratin and CD31 negative, demonstrating that some donor‐derived stem cells differentiated into endometrial stromal cells but not epithelial cells or vascular endothelial cells.

## Discussion

The endometrium of mammals undergoes regeneration in each menstrual cycle. Endometrial stem cells residing in the basalis layer are considered to act as a source for endometrial renewal. Bone marrow stem cells have been reported to differentiate into various cell types as endodermal, mesodermal and ectodermal original cells, including endometrial cells. However, there have been no studies comparing the recruitment capacity of these two kinds of stem cells to the endometrium; there is no report comparing systemic administration and local intra‐luminal uterine injection for these cells injected, either. In this study, we show that systemic administration route results in greater recruitment of donor cells to the injured uterus than direct local injection. Furthermore, we demonstrate that BMDCs are recruited to the uterus in higher numbers than UDCs.

In this study, we used a mouse model consisting of a mild endometrial injury, which did not result in unwanted uterine adhesions, but was sufficient to stimulate increased recruitment of donor cells to the uterus. This models therapeutic uterine injury (endometrial scratch), which has been increasingly utilized in an attempt to improve endometrial receptivity and implantation in patients with recurrent implantation failure and/or thin endometrium 20, 21. Following uterine injury and BMDCs/UDCs injection, GFP^+^ cells were detected in the uterus of the treated mice. There were no differences between the injured and non‐injured horn in terms of GFP^+^ cell recruitment, suggesting that uterine injury provides a stimulus for recruitment of BMDCs/UDCs to the endometrium globally and not specifically to the site of injury. This result is consistent with our previous study in which a more pronounced uterine injury recruited BMDCs to the endometrial stroma of both uterine horns equally following IV injection 22. We also have shown that BMDCs recruitment to uterus is increased in response to ischaemia/reperfusion injury regardless of the existence of an oestrus cycle or hormonal stimulation 18. The recruitment of BMDCs/UDCs to the endometrium following injury may be the result of local inflammation as part of a tissue repair mechanism 22.

In the present study, the percentage of donor cells engrafted in the uterus was relatively low, ranging from 0.02% to 0.5%. In a study of murine model of Asherman's syndrome from our laboratory, in which the injury is more serious than the model presented herein, the Y chromosome bearing CD45‐donor cells represented less than 0.1% of total endometrial cells 22, comparable to our model. In a previous study, BMDCs showed long‐term engraftment for 8 months in mice with less numbers (about 42/100,000) of uterine stromal cells 18. In addition, human damaged uterus as Asherman's syndrome can repair the uterine cavity with little remaining endometrium 25. Santamaria *et al*. reported that CD133^+^ bone marrow‐derived stem cells improved tissue regeneration in Asherman's syndrome patients 23. The same group showed in a related study that the same patients' CD133^+^ BM cells are recruited to the uterus following systemic injection in an immunodeficient Asherman's mouse model, representing about 0.6% of total uterine cells 24. These indicate that the injured uterus can be repaired even with relatively little exogenous stem cells recruited to the uterus. It remains to be determined whether strategies to enhance the number of engrafted stem cells could improve endometrial tissue repair.

In this study, some GFP^+^ cells were vimentin positive at 3 weeks after BMDCs/UDCs injection, suggesting that donor cells were not only recruited to the endometrium but also differentiated into stromal cells. Moreover, CD45 was used to distinguish endometrial cells from transient leucocytes recruited in the endometrium. The percentage of CD45‐negative GFP+ cells in both groups was high, suggesting that most of the graft‐derived cells became resident non‐leucocyte cells in the endometrium. We could not detect donor‐derived endothelial or epithelial (luminal or glandular) cells. Similar findings were reported by our group which evaluated uterine BMDCs at 1 month post‐transplant in a non‐gonadotoxic bone marrow transplant model 26. In another study from our laboratory which evaluated a later time point post‐transplant, both stromal and epithelial cells of BM origin were detected at 8 months after BMDCs transplantation 18. Others also showed that BMDCs were detectable only in the stromal compartment at 3 month post‐transplant and were only detected in the glandular or luminal epithelial compartments after 12 months post‐transplant 27. Taken together, these data suggest that the stroma is the primary and main target for stem cell recruitment and uterine tissue regeneration following injury.

Injection of BMDCs led to higher percentage of donor cells in the uterus compared with UDCs injection. The number of BMDCs injected is usually 1 × 10^7^ cells 18, 22, 28. However, for UDCs injection, there has been no report about the number of UDCs injected systemically. In this study, we performed a preliminary experiment to determine a safe systemic dose and tried several doses by tail vein injection. The survival rate post‐injection following 1 × 10^6^ uterine cells injection was diminished, while the survival rate for 5 × 10^5^ uterine cells injection was high. Mortality using the high dose of UDCs was observed in close proximity to injection, suggesting that embolic event occurred when number of uterine cells injected exceeds a certain threshold.

Human endometrial stem cell populations have been isolated and differentiated into endometrial glandular epithelial, stromal and endothelial cells *in vitro* and in immunodeficient mouse models 3, 4, 5, 6, 29. Our study is the first proof‐of‐concept that endometrial stem cells may be used therapeutically to repair the uterus, providing important information regarding suitable number of cells to inject and route of administration, which may inform investigators developing endometrial stem cell‐based therapies.

Bone marrow‐derived stem cells have been reported to not only differentiate into all types of haematopoietic lineage cells, but also differentiate into various nonhematopoietic tissue cells such as endodermal, mesodermal and ectodermal 30, including various mature endometrial cells 16, 31, 32, 33, 34. Nevertheless, most studies of the differentiation potential of endometrial derived stem cells have focused on mesodermal differentiation, for instance, differentiation into adipocyte 7, 35, osteocytes 36, chondrocytes 8, smooth muscle cells 37 and fibroblasts 9 *in vitro*. These indicate that endometrial‐derived stem cells have extensive mesodermal multipotency. Some studies showed they have the capacity for differentiation into endodermal cells such as pancreatic lineages 38, 39, hepatocyte‐like lineage 40, ectodermal lineage dopaminergic neuron‐like cells 41. Further studies are necessary to investigate the differences in therapeutic potential and mechanisms between BMDCs and UDCs in repairing the uterus.

Most previous studies investigating BMDCs recruitment to the uterus administered BMDCs systemically by either tail vein injection 22, 27, 28, 32, 42, 43 or internal jugular vein injection 18. Recently, human BMDSCs obtained from 10 patients with refractory AS or endometrial atrophy were separately injected to 10 NOD‐SCID mice either intrauterine or tail vein 24. However, no conclusion was reached regarding superiority of either route for BMDC injection. Our findings indicate that systemic injection of BMDCs/UDCs results in greater recruitment of GFP^+^ cells to the uterus as compared to local injection. Our findings that stem cells were recruited to stroma but not epithelium, and appeared in close proximity to blood vessels but not in the vascular wall suggest that the stem cells get to the uterus *via* blood vessels. Similar findings were reported by Cervello *et al*. 24 following systemic BMDCs injection. When BMDCs/UDCs are injected systemically, the blood provides them with various trophic factors which may enhance their survival as compared to intra luminal local injection. This may explain why stem cells injected locally have lower percentage of GFP^+^ in the uterus and decrease over time. It would be interesting to explore whether the use of scaffold with trophic factors may enhance survivability in the uterine cavity and engraftment of the cells.

In conclusion, systemic route of administration of BMDCs or UDCs results in better recruitment to the injured uterus than local injection. In addition, BMDCs may be more suitable for restoring the injured uterus than UDCs. These findings may inform investigators developing stem cell‐based therapies targeting the uterus.

## Conflict of interest

All authors declare no conflict of interest.
